# A Simultaneous Concept Analysis to Provide Clarity Between Obstetric Violence and Birth Trauma

**DOI:** 10.1111/birt.70019

**Published:** 2025-09-12

**Authors:** Kripalini Patel, Liz Newnham, Kathrine Gillett, Allison Cummins

**Affiliations:** ^1^ School of Nursing and Midwifery University of Newcastle Callaghan New South Wales Australia; ^2^ College of Nursing and Health Sciences Flinders University Adelaide South Australia Australia

**Keywords:** birth trauma, disrespect and abuse, mistreatment, obstetric violence, psychological birth trauma, traumatic childbirth

## Abstract

**Background:**

In perinatal care, obstetric violence and birth trauma are two distinct yet often conflated concepts. This confusion can obscure the specific harms of obstetric violence, as its impact is frequently subsumed under the broader idea of birth trauma, leading to underreporting of obstetric violence. Simultaneous concept analysis is used to clarify two related concepts by comparing their unique elements and identifying overlaps.

**Aim:**

To compare the antecedents, attributes, and consequences of both the concepts and to identify their intersections.

**Methods:**

A comprehensive search across PubMed, Google Scholar, CINAHL, and ProQuest yielded 98 articles on obstetric violence and 62 on birth trauma. Thematic analysis of antecedents, attributes, and outcomes informed a comparative validity matrix.

**Results:**

Obstetric violence and birth trauma have different causes and characteristics but lead to similar outcomes. Birth trauma arises from experiences like fear or unmet expectations, while obstetric violence involves abuse by providers and systemic failures. Both result in emotional distress, anxiety, and fear of future childbirth.

**Conclusion:**

Existing literature uses the term “birth trauma” as a euphemism for what is essentially obstetric violence. Considering the conceptual confusion between the subjective trauma arising from childbirth experiences and the trauma specifically resulting from abuse by healthcare providers, we are suggesting a new term, “Obstetric Trauma” This would specifically indicate the structural and institutional consequences of obstetric violence on women. It would also help guide targeted interventions, policy changes, and support systems aimed at preventing obstetric violence and promoting respectful maternity care.

## Graphical abstract

There are critical gaps and conceptual confusion between the subjective trauma arising from childbirth experiences (birth trauma) and the trauma specifically resulting from abuse, coercion, and neglect by healthcare providers (obstetric violence); we propose a new term, “obstetric trauma” Obstetric trauma would specifically indicate the consequences of obstetric violence resulting from mistreatment and coercion. This term would not only help distinguish it from birth trauma but also emphasize the systemic and avoidable nature of trauma.

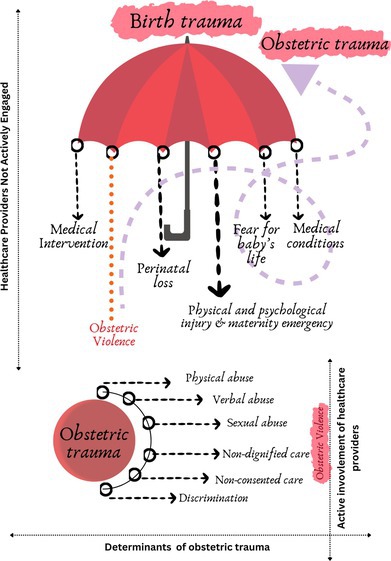

## 1 Introduction

In the context of perinatal care, two critical yet often conflated concepts are obstetric violence and birth trauma. The intersection of these two concepts presents a significant area for exploration within maternal healthcare because although both concepts have profound implications distinctly for the well‐being of birthing people, confusing the two may lead to under‐reporting the impact of obstetric violence as its impact is often subsumed into the concept of birth trauma.

### 1.1 Obstetric Violence

Obstetric violence is recognized as a specific form of violence against those who give birth that extends beyond obstetric complications. It may be perpetuated by anyone who stands with the woman giving birth, including doctors, midwives, nurses, and traditional birth attendants. It involves mistreatment, coercion, and disrespect during birth because of systemic power imbalances and discriminatory practices [1, 2, 3]. Venezuela became the first country to legally recognize obstetric violence as a punishable offense under its laws protecting birthing people from violence [4]. The United Nations has also classified obstetric violence as a form of gender‐based violence [5]. To understand the concept of obstetric violence, it is important to understand the underlying factors that contribute to the under‐reporting of this issue [6]. Factors include a lack of awareness of human rights during childbirth, normalization of disrespect and abuse, fear of not receiving treatment if complaints against healthcare providers are raised, and a lack of accessible reporting mechanisms at the health system level [7]. The under‐reporting of obstetric violence exacerbates these issues and allows disrespect and abusive practices to continue unaddressed within healthcare systems. When birthing people unable to voice their experiences of disrespect or abuse, the mistreatment remains hidden. This silence has far‐reaching consequences not only on their health and that of their newborns but also at a broader societal level [8].

Researchers worldwide have investigated the potential causes, nature, and effects of obstetric violence. However, there is limited literature available that comprehensively examines its impact on birthing people, their newborns, and society at large. For example, Schroll et al. (2013) reported that birthing people who experience obstetric violence may face difficulties in their sexual relationships with their partners [9]. Some birthing people even consider selecting caesarean sections in future pregnancies to avoid violence during childbirth [10], and in some cases, they delay seeking facility‐based care during complications because of fear of mistreatment [11]. The experience of obstetric violence can also result in postpartum depression or Post Traumatic Stress Disorder (PTSD), as birthing people may feel out of control and traumatized during childbirth [12]. Existing research often fails to clearly distinguish between obstetric violence and birth trauma. When discussing the impact of obstetric violence, studies tend to refer broadly to traumatic childbirth or birth trauma, rather than isolating the specific impact of obstetric violence itself. While early research into obstetric violence identified its typologies [13], nature [14, 15], and determinants [11, 16], the consequences of the impact of obstetric violence as a distinct concept to birth trauma have not yet been studied explicitly.

### 1.2 Birth Trauma

The second concept this paper focuses on is that of birth trauma, a concept which can involve both clinical and non‐clinical attributes. Beck (2004) highlighted that birth trauma is highly subjective, defined by the birthing person's perception [17]. It is often considered to be caused by a severe birth injury or complication to the mother or the injury/death of her infant [18]. Clinical causes of birth trauma may include interventions or emergencies, such as caesarean section, pre‐term birth, postpartum haemorrhage [19] while non‐clinical causes can include lack of support, fear for her own and the baby's life, unwanted pregnancy, and episodic experiences of obstetric violence [20]. It can negatively affect the mother's/parent's mental health [21], parent‐infant relationships [22], and future reproductive choices [18]. It can be linked to PTSD [23] and suicide, a leading cause of maternal death, recorded as 5%–18% of all maternal deaths in high‐income countries [24].

Various terms, such as traumatic birth [25], traumatic childbirth [26], negative childbirth experience [27] and difficult delivery [28], have been used to describe childbirth experiences that result in some sort of psychological dysfunction or trauma. However, birth trauma appears to be an umbrella term that includes both clinical experiences that are perceived as negative or frightening in themselves and experiences where health care providers have been directly involved in causing harm.

There are concerns related to using the term birth trauma to describe the violence and abuse towards birthing people during birth, and there is a lack of evidence that can distinguish between obstetric violence and traumatic birth [29]. Birth trauma is often regarded as a subjective experience, closely tied to an individual's personal feelings and perception about the childbirth process and its outcome. Whereas obstetric violence is a violation of human rights and more insidious, typically involving power imbalances, coercion, and hierarchical dynamics where healthcare providers exert control over birthing people, often disregarding their autonomy and bodily integrity. It is significant to understand that not all traumatic births involve obstetric violence. Even when birthing individuals receive the highest standard of healthcare, they may still experience trauma due to an unexpected clinical situation. It is a subjective experience. Also, when abusive care occurs, obstetric violence can become the direct trigger of a traumatic birth experience, creating a zone of intersection between the concepts. In essence, obstetric violence is an act usually perpetrated by healthcare providers or systems, whereas birth trauma is an outcome or experience for the birthing people.

Academic discussions which categorize obstetric violence within the broader concept of birth trauma potentially conceal the unethical practices of healthcare providers by framing its effects as a part of a more generalized birth trauma discourse. Concealment creates a zone of intersection between the concepts which highlight a critical gap in existing literature. Understanding this intersection is essential because obstetric violence, when experienced during childbirth, may directly contribute to traumatic birth experiences, blurring conceptual clarity. There is a need to specifically acknowledge the existing problem of obstetric violence to counteract it and work towards respectful perinatal care for all. As researchers suggest, in high‐income countries, there is often a failure to acknowledge the problem of obstetric violence [30].

The aim of this Simultaneous Concept Analysis is to distinguish the unique characteristics, causes, and consequences of each concept, as well as to identify their points of intersection. This approach is crucial for developing clearer definitions, creating more targeted interventions, and improving communication between healthcare providers and birthing people.

## 2 Methods

A Simultaneous Concept Analysis was employed to explore the similarities and differences between obstetric violence and birth trauma [31]. Developed by Haase et al., Simultaneous Concept Analysis is grounded in Rodgers' evolutionary concept development theory [32] and is particularly suitable when analyzing concepts that are closely related yet distinct as is the case with obstetric violence and birth trauma. The methodology consists of nine stages and allows for the clarification and refinement of related concepts. A detailed description of the nine steps is provided in the Supporting Information.

### 2.1 Data Sources

A comprehensive literature search was conducted across multiple databases, including PubMed, Google Scholar, CINAHL, and ProQuest. The search terms “obstetric violence”, “mistreatment”, “disrespect and abuse”, “psychological birth trauma”, “traumatic childbirth” and “birth trauma” were used to identify relevant studies in each database. The detailed search strategy is provided in a Supporting Information. No time or location restrictions were applied to the search, as our goal was to establish a robust conceptual foundation for both obstetric violence and birth trauma by retrieving as much relevant information as possible. This approach ensured we captured the evolution of these concepts over time, allowing for a more thorough analysis.

Articles were included if they met the following criteria: (1) published in peer‐reviewed scholarly journals, (2) available in English, and (3) the terms “obstetric violence” or “birth trauma” appear in either the title or abstract. By applying these inclusion criteria, we focused on the most relevant literature that directly addresses the conceptualization and analysis of these terms in a healthcare context.

**FIGURE 1:**
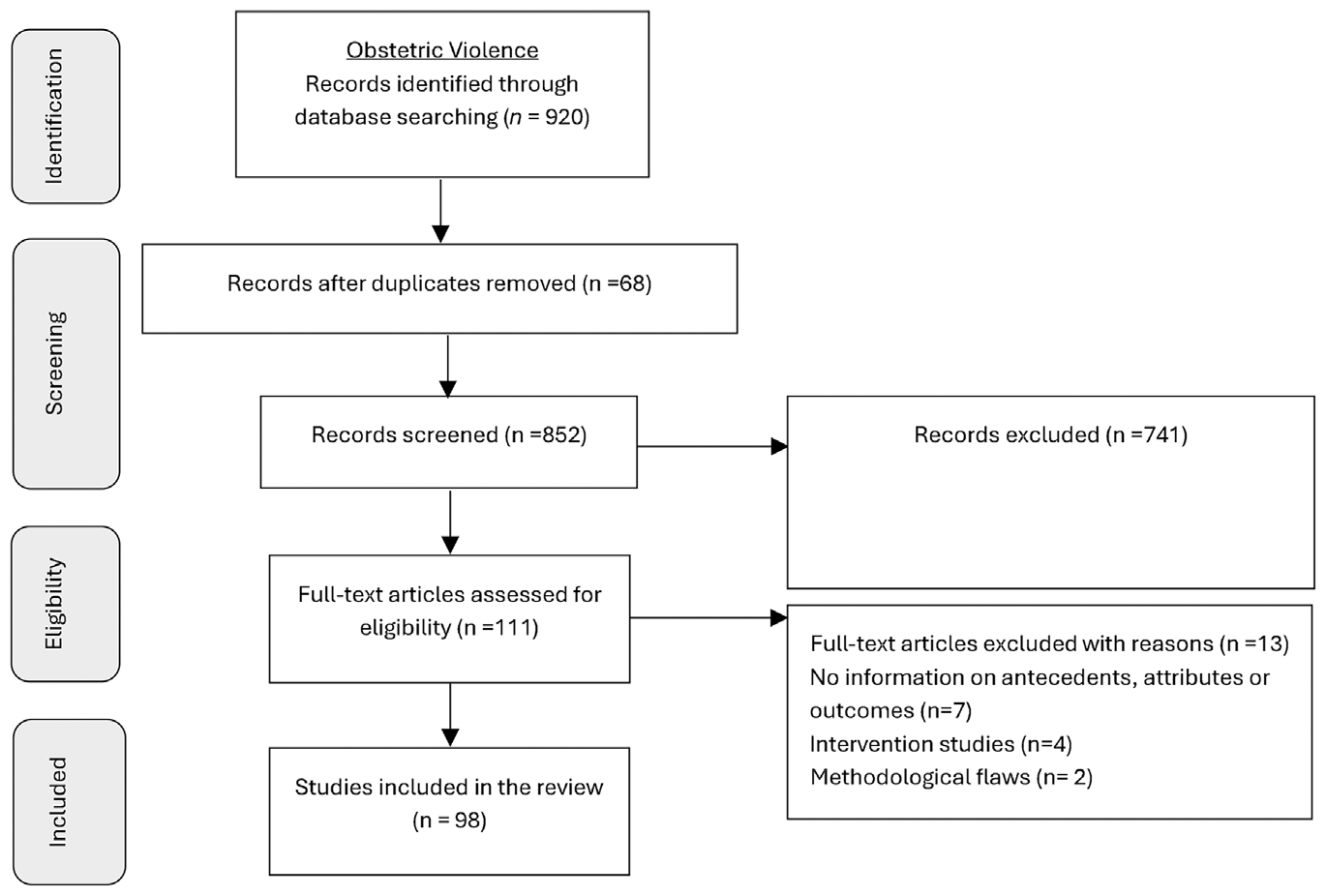
PRISMA flow diagram for obstetric violence.

**FIGURE 2:**
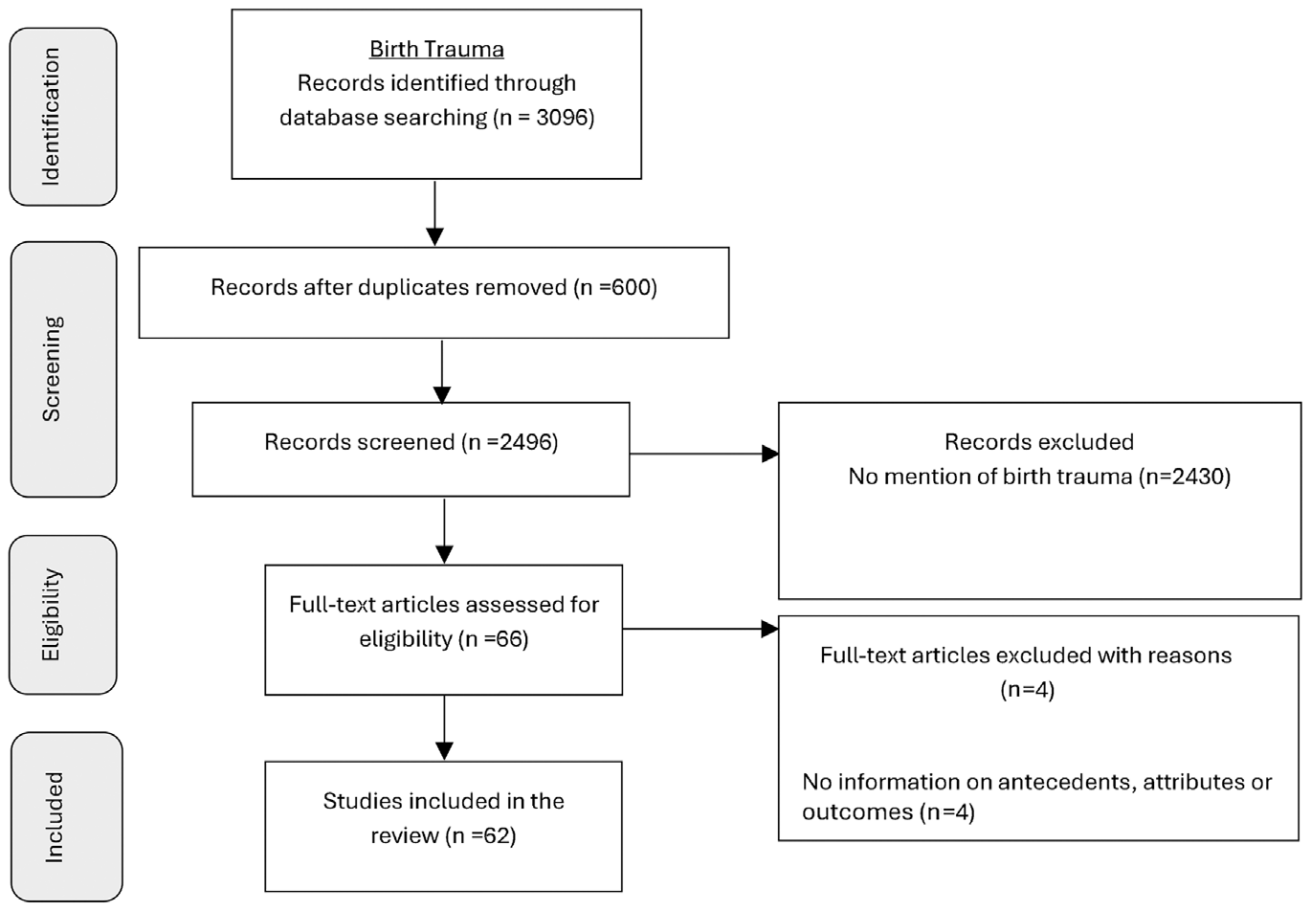
PRISMA flow diagram for birth trauma.

A total of 920 articles were identified specifically about obstetric violence, and 3096 about birth trauma. After screening the title and abstract, 98 articles on obstetric violence and 62 articles on birth trauma were included in the SCA. PRISMA flow diagram for each concept is provided in Figure 1 and Figure 2 respectively.

### 2.2 Data Analysis

The aim of data analysis is not to create a final, complete definition of the concepts, rather to offer a starting point for further conceptual exploration [32]. Following this approach, thematic analysis was conducted, and each article was reviewed as many times as needed in order to identify the process model− attributes, antecedents, and outcomes of each concept before constructing the validity matrix [32]. The process involved multiple cycles of coding and recoding to ensure a rigorous and systematic process. Peer debriefing sessions were also held to validate the findings. In the following phase, critical attributes, theoretical definitions, antecedents, and outcomes were independently identified for each concept, then compared to inform the validity matrix (Table 3).

## 3 Results

The results are presented individually to allow a complete understanding of the unique and individual approach to each concept. A total of 92 articles were included under the concept of obstetric violence, and 62 under birth trauma. Included articles were provided in the Supporting Information. For each of these concepts, a definition, antecedents, attributes, outcomes, and surrogate terms are discussed below.

### 3.1 Concept 1: Obstetric Violence

#### 3.1.1 Definitions

This literature review identified six definitions across different terminology around obstetric violence. As Perez (2010) stated, “overmedicalisation of childbirth is seen as leading to dehumanisation resulting in a loss of autonomy” [4]. Further, individual acts of disrespect and abuse have been described such as slapping or scolding, in addition to structural issues such as overcrowding and unhygienic conditions [33]. In 2017, Chadwick provided a definition that focused on how societal norms around race, class, and gender influence the practice of obstetric violence as they demand birthing people to demonstrate a “good birthing body” [2]. Sen (2018) reported that violations of dignity and well‐being are based on discriminatory biases, which may result in intentional or systemic harm among birthing people [34]. Miltenburg (2018) provided a definition which can be used to understand how power, hierarchy and control can bring obstetric violence among birthing people [35]. Sadler (2016) stated obstetric violence is a form of systemic violation of birthing people's rights and autonomy in childbirth [36]. And lastly, a recent definition by Garcia (2020) focused on the context of the United States and highlighted that birthing people can experience obstetric violence by healthcare providers during any stage of the childbearing process such as invasive procedures performed without informed consent, under coercion, or against their refusal [37]. The process model for the concept of obstetric violence is provided below (Table 1).

**TABLE 1:** Process model of obstetric violence.

Obstetric violence	Characteristics
Antecedents^(8,15,44–89)^	1. Demographic and socioeconomic factors – Younger maternal age (especially under 19) – Lower socioeconomic status (e.g., low income, poverty) – Limited education or no access to childbirth preparedness programs – Rural residence or marginalized communities (e.g., lower caste, disadvantaged ethnicity) – Non‐majority religious affiliation (e.g., Muslim women) – Unmarried status or lack of family support – Women with multiple previous births (higher parity) – LGBTQ+ identification or gender‐related inequities 2. Healthcare delivery factors – Public sector or low‐resource facilities with poor hygiene and infrastructure. – Inadequate staffing and lack of accountability systems – Denial of labour companions or chosen support during childbirth – Prolonged hospital stays, delays in care, and long labour duration – Procedural gaps, including absence of informed consent and unmet care preferences – Lack of pain management, privacy, or respectful communication 3. Provider‐related factors – Verbal abuse (shouting, blaming, derogatory remarks) – Physical abuse (slapping, pinching, or forced practices like sterilization) – Psychological violence (intimidation, humiliation, and neglect) – Lack of professional training, supervision, and awareness of respectful care – Stress, burnout, and hierarchical dynamics within healthcare teams – Cultural insensitivity and discriminatory attitudes 4. Systemic and institutional factors – Weak health systems with resource constraints and mismanagement – Poor facility environments (e.g., overcrowding, lack of supplies, dirty wards) – Restrictive policies (e.g., no birth companions allowed, non‐adherence to patient rights) – Inequitable or hierarchical decision‐making processes – Limited legal mechanisms for redress and accountability 5. Cultural and social norms – Normalization of violence and disrespect during childbirth – Gender inequities and harmful myths about pain tolerance or obedience – Marginalization of women and dehumanization in care practices – Stigma and discrimination based on race, class, or religion 6. Maternal and pregnancy‐related factors – Complications during delivery or need for emergency procedures (e.g., caesarean sections) – Unplanned or emergency caesarean births – Absence of antenatal care visits or birth plans not respected – Newborn health concerns (e.g., neonatal intensive care admission) 7. Healthcare context and policies – Facility‐based births in under‐resourced public hospitals – Overworked staff and insufficient provider‐to‐patient ratios – Normalization of abusive practices by senior staff or within institutional cultures – Lack of integration of traditional practices with biomedical care approaches
Attributes^(5,17,51,52,65,67,69,74,80,83,86,87,89–113)^	1. Abuses among women – Physical Abuse: Slapping, hitting, pinching, beating, fundal pressure, unanaesthetised procedures, episiotomies without consent – Verbal Abuse: Shouting, scolding, insults, judgmental comments, threats, and derogatory remarks toward patients or their relatives – Psychological Abuse: Ignoring or dismissing women's needs, judgmental attitudes, lack of supportive communication, and intentional neglect – Sexual Abuse: Non‐consensual or inappropriate pelvic examinations and invasive procedures
	2. Neglect and abandonment – Being left alone during labour or ignored – Failure to respond to requests for help – Neglect during prenatal and peripartum care 3. Violations of autonomy and consent – Non‐consented care (e.g., invasive interventions, surgical operations, and procedures) – Stripping patients of autonomy in decision‐making – Coercion and bullying 4. Discrimination – Stigma based on race, class, or social status – Discriminatory care or denial of care 5. Lack of professional standards – Failure to provide pain relief – Poor hygiene, lack of cleanliness, and substandard care – Denial of birth companions – Refusal or delay of skilled care 6. Breaches of privacy and confidentiality – Lack of privacy during procedures or care – Non‐confidential handling of patient information 7. Systemic issues – Power dynamics favoring healthcare providers over patients – Inadequate infrastructure and staffing in healthcare facilities – Detainment of patients for non‐payment of fees or abandonment by relatives – Provider demotivation and reliance on informal payments
Outcomes^(23,53,60,65,73,85,108,114–129)^	1. Psychological impacts on women – Increased risk of postpartum depression (PPD) – Post‐traumatic stress disorder (PTSD) – Long‐term psychological effects, including emotional distress, self‐doubt, and fear of future childbirth – Immediate distress and long‐term emotional impact – Feelings of distrust, hatred, and psychological distance towards healthcare providers 2. Maternal and neonatal health impacts – Maternal injuries and complications – Neonatal injuries and related complications – Difficulties in exclusive breastfeeding 3. Impact on Healthcare Utilization – Reduced attendance at postpartum and childcare consultations – Decreased and delayed use of postnatal health services for both women and newborns – Reduced intention to use healthcare facilities for future births – Decreased willingness to recommend healthcare facilities to others – Deterrence from seeking institutional care for future deliveries 4. Social and Emotional Consequences – Erosion of trust between women and healthcare providers – Adverse impact on women's dignity and autonomy during childbirth – Increased visibility and normalization of obstetric violence in childbirth practices 5. Impacts on Healthcare Professionals – Professional dissatisfaction and emotional burden for midwives 6. Broader Health System Impacts – Increased health inequities – Complex barriers to accessing sexual and reproductive healthcare – Deprivation of respectful and informed care during childbirth

#### 3.1.2 Surrogate Terms

**FIGURE 3:**
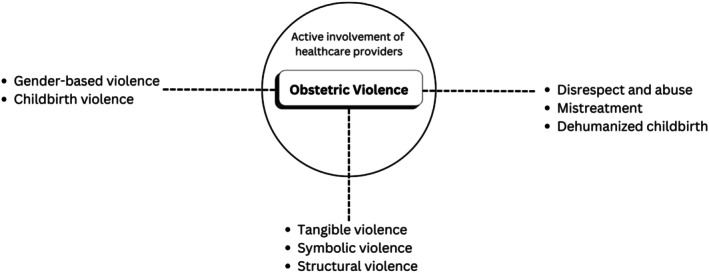
Conceptual landscape of obstetric violence terminology.

The terminologies related to obstetric violence seem to have a different terminological spectrum, used by different researchers based on their subjectivity. We present a conceptual landscape of obstetric violence terminology below (Figure 3).

### 3.2 Concept 2: Birth Trauma

#### 3.2.1 Definitions

An extensive range of studies have examined traumatic birth; despite this, a clear definition of a traumatic birth has not been agreed upon within the literature. In 2010, Elmir et al. stated that there is no consistent definition of traumatic birth and no systematic way to assess birth trauma, and the terms birth trauma and traumatic birth are used frequently and synonymously [38]. However, researchers have proposed definitions by analyzing the underlying factors and nature of traumatic birth. Beck (2004) defined a traumatic birth event as one during labor and birth where the mother/parent or her infant faces actual or threatened severe injury or death, leading the mother to feel intense fear, helplessness, loss of control, and horror [17]. Similarly, Greenfield et al. (2016) reported that a traumatic birth occurs when a mother/parent feels deeply distressed or disturbed by the events, any physical injury, or the care received during childbirth, with this distress having a lasting impact [39]. Leinweber et al. (2022) further defined a traumatic birth experience as a woman's/parent's experience of birth‐related interactions or events that provoke intense distress, resulting in both immediate and lasting negative effects on their health and wellbeing [40]. Most recently, Sun et al. (2023) described psychological birth trauma as a woman's personal experience of distress linked to birth, surrounded by intense emotional pain throughout the birth to the postpartum phase, which impacts the mother/parent both negatively and positively [41]. The process model for the concept of birth trauma is provided below (Table 2).

**TABLE 2:** Process model of birth trauma.

Birth trauma	Characteristics
Antecedents^(134–158)^	1. Childbirth complications – Avoidable and unavoidable childbirth complications – Emergency caesarean sections – Mode of delivery: caesarean, instrumental, and operative vaginal births – Manual placenta removal and high labour pain – Lack of pain relief during labour 2. Maternal health and mental health factors – Pre‐existing conditions (e.g., depression, PTSD), past trauma, history of sexual or partner violence – Mental health challenges related to childbirth – Pregnancy complications and poor health – Inadequate prenatal care, low exercise, unplanned pregnancies – Stress, feelings of powerlessness, and lack of partner support. 3. Healthcare experience and provider interactions – Poor medical interactions, lack of control during childbirth, dissatisfaction with social support – Negative interactions with healthcare providers (e.g., prioritization of provider agenda, dehumanized treatment) – Dysfunctional maternity care systems and provider violations 4. Infant and family factors – Infant complications (e.g., NICU placement, preterm delivery) – Maternal–infant attachment, couple's relationship, and parenting self‐efficacy issues 5. Socio‐demographic and environmental factors – Socio‐demographic factors: maternal age, parity, migration background, and assistance during labour – Cultural beliefs and family income – Human and non‐human environmental factors affecting birth 6. Subjective experience and perception – Subjective and perceived traumatic birth experiences – Fear of childbirth and negative birth environment
Attributes^(20,32,133,134,159–168)^	1. Feelings of control and empowerment – Feelings of lack of control, helplessness, and despair – Attempts to reclaim autonomy, empowerment, and pursue healing in childbirth – Fear and mistrust of healthcare providers, seeking information to feel in control 2. Mental health challenges – Depression, fear of childbirth, and postpartum depression – Persistent emotional distress originating from the birth process into postpartum – Pre‐existing health issues compounded by birth‐related stressors 3. Perception of childbirth and trauma – Perception of childbirth as traumatic and subjective feelings of trauma – Discrepancy between birth expectations and reality, physical pain, and fear for safety 4. Feelings of isolation and disconnection – Feelings of isolation and disconnection from healthcare providers – Powerlessness and exclusion during childbirth 5. Postpartum stressors and recovery – NICU/SCN parenting stress, difficult recovery, and postnatal stressors – Unmet birth expectations and challenges in recovery
Outcomes^(25,31,148,158,163,169–187)^	1. Mental health consequences – PTSD, depression, anxiety, and fear of childbirth following traumatic birth experiences – Psychological birth trauma, including nightmares, flashbacks, and emotional distress 2. Impact on maternal–infant bonding and parenting – Negative effects on maternal–infant bonding, including distress in parenting and bonding challenges – Impact on breastfeeding, including reduced breastfeeding duration 3. Family and social effects – Strained couple relationships and negative effects on family and social roles – Negative impact on self‐identity, psychological well‐being, and social isolation 4. Reproductive and health outcomes – Reduced subsequent childbearing or extended intervals between pregnancies – Physical and psychological health issues, including lower health‐related quality of life and increased healthcare usage 5. Anniversary reactions and trauma recurrence – Anniversary reactions and recurrence of trauma 6. Distress from unmet expectations – Distress from unmet expectations, family neglect, and fear during childbirth

#### 3.2.2 Surrogate Terms

**FIGURE 4:**
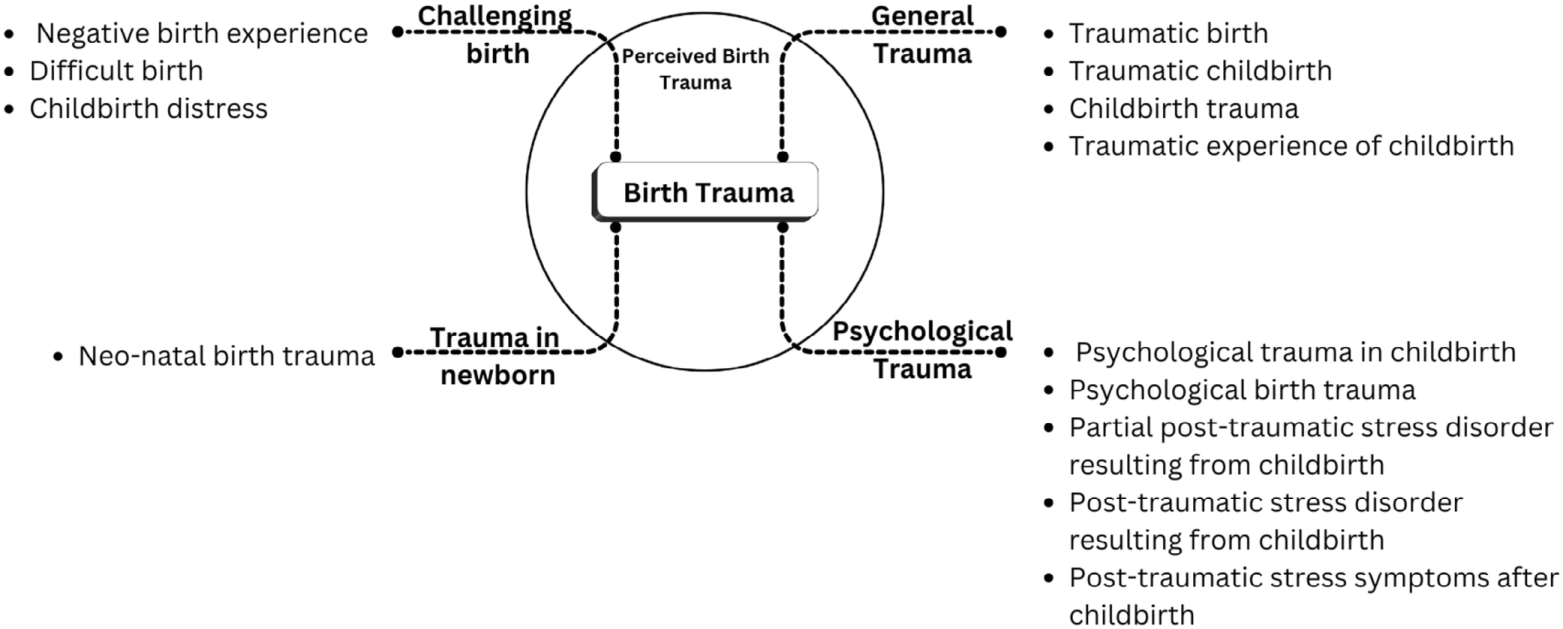
Conceptual landscape of birth trauma terminology.

The terminologies related to birth trauma appear to have a different terminological spectrum, which is used by different researchers based on their subjectivity. We present a conceptual landscape of birth trauma terminology below (Figure 4).

#### 3.2.3 Validity Matrix for Antecedents, Attributes and Outcomes of Birth Trauma and Obstetric Violence

**FIGURE 5:**
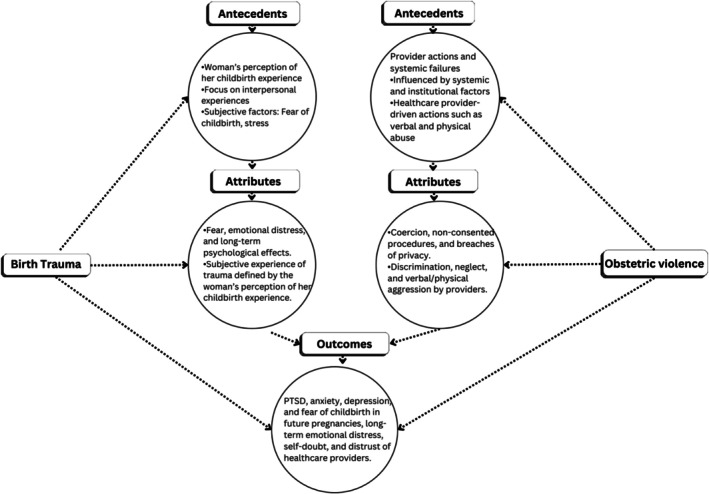
Graphical presentation of validity matrix.

The below validity matrix highlighted the comparisons between obstetric violence and birth trauma (Table 3). The graphical presentation is provided in Figure 5.

**TABLE 3:** Validity matrix for birth trauma and obstetric violence.

Category	Birth trauma	Obstetric violence
Antecedents	Source: Woman's perception of her childbirth experience, including fear, unmet expectations, and distress	Source: Provider actions and systemic failures, including abusive, disrespectful, or negligent care practices
	Healthcare System Role: Indirectly linked to the healthcare system, focusing on interpersonal experiences and perceived lack of support	Healthcare System Role: Directly influenced by systemic and institutional factors like understaffing, overcrowding, and poor infrastructure
	Subjective factors: Fear of childbirth, stress, unmet expectations, and pre‐existing mental health issues (e.g., PTSD, anxiety)	Healthcare provider‐driven actions: Verbal, physical, or psychological abuse; neglect; systemic disrespect; lack of informed consent
	Healthcare experience: Lack of control, lack of communication, disconnection from providers, inadequate pain management	Systemic issues: Understaffing, overcrowding, and lack of accountability in healthcare facilities
	Social and cultural factors: Stigma, negative family dynamics, and societal expectations	Cultural normalization: Gender inequities, and discrimination based on race/class
Attributes	Perceived lack of control: Helplessness, powerlessness, and isolation	Direct violations of autonomy: Coercion, non‐consented procedures, and breaches of privacy
	Nature: Fear, emotional distress, and long‐term psychological effects	Nature: Intimidation, neglect, and verbal/physical aggression by providers
	Subjective experience: Trauma defined by the woman's perception of her childbirth experience	Objective provider actions: Disrespect, abusive behavior, and systemic neglect impacting care quality
	Family and Partner Support: Less dependent on family or partner support	Family and Partner Support: Strongly influenced by the presence or absence of family/partner support during labour and delivery
Outcomes	Mental health effects: PTSD, anxiety, depression, and fear of childbirth in future pregnancies	Mental health effects: Long‐term emotional distress, self‐doubt, and distrust of healthcare providers, PTSD, anxiety, depression, and fear of childbirth in future pregnancies
	Impact on maternal–infant bonding: Challenges in breastfeeding, attachment, and parenting confidence	Impact on maternal–infant bonding: Poor infant outcomes due to inadequate or harmful practices, challenges in breastfeeding
	Healthcare avoidance: Hesitancy or refusal to use institutional care for subsequent births	Healthcare avoidance with systemic repercussions: Reduced utilization of health services and diminished trust in healthcare institutions
	Social impacts: Strained family relationships, social isolation, and disrupted self‐identity	Healthcare inequities: Increased barriers to respectful care

## 4 Discussion

This simultaneous concept analysis focused on two interrelated concepts, obstetric violence and birth trauma, with particular emphasis on their attributes, antecedents, and outcomes. This analysis widens the lens of both concepts and clarifies that while antecedents and attributes are different for both concepts, the outcomes are similar. This further justifies that when obstetric violence is considered under the umbrella of birth trauma, we lose an opportunity to recognize and act on it.

Confusion often arises between the impact of obstetric violence and birth trauma. When birth happens in an abusive environment, this is often considered birth trauma too; however, we need to understand that birth trauma is a personal perception and can be felt and experienced despite receiving respectful maternity care [38]. Obstetric violence, on the other hand, involves mistreatment, coercion, and disrespect during childbirth because of systemic power imbalances and discriminatory practices—a distinction that is often lost within multiple definitions [2].

Few researchers have identified the differences between obstetric violence and birth trauma or discussed the points of intersection. For example, Darilek et al. (2018) acknowledged the clear overlap between birth trauma and obstetric violence; emotional birth trauma is a potential outcome of obstetric violence and suggested the need to explore the connection between the two issues [42]. Further, Salter et al. (2023) found that clinicians' descriptions of birth trauma were frequently mapped onto established mistreatment categories even without prompting. In over 30 h of clinician interviews about “birth trauma,” the word mistreatment was seldom used despite being described as numerous mistreatment events (e.g., performing procedures without consent or neglecting non‐English‐speaking patients) as part of traumatic birth narratives. Such findings imply that some healthcare professionals may use “birth trauma” as a euphemism for what is essentially obstetric violence [43]. These findings are quite consistent with the antecedents of both the concepts in this study. Our analysis found that certain antecedents—notably systemic neglect, poor communication, and absence of informed consent appeared in birth trauma literature which classified as the types of obstetric violence, suggest that the boundary between the two concepts can sometimes blur in practice. While asking birthing people what contributes to their birth trauma, the major responses were, inattentive or even hostile treatment by healthcare personnel, lack of consent, inadequate information, and dissatisfaction with maternity [43]. Birth trauma was explored in high income countries and categorized birthing people's narratives' of obstetric violence as “subjective distress” [44] and “interaction hotspots” [45]. While maternal birth trauma is strongly influenced by “negative health care provider interactions and dysfunctional operation of the maternity care system,” [46] alongside limited informed decision‐making and support, all of which contribute to birthing people feeling powerless [47], these are precisely the conditions that define obstetric violence. Thus, although obstetric violence and birth trauma originate from different theoretical frameworks (human rights violation vs. psychological trauma), their shared antecedents indicate a substantive intersection in lived experience.

Existing literature also highlights the increased prevalence of “guilt”, “powerlessness”, “post‐traumatic stress disorder” and “moral injury” among healthcare professionals when they unable to provide quality healthcare to birthing people [48]. Reports of Secondary traumatic stress has been reported among nurses providing intrapartum care and witnessing obstetric violence towards birthing people by other healthcare providers [49]. Such context may position healthcare professionals in a place where they might perpetuate violence among birthing people without any intention [50]. Protecting their autonomy, mental health, and ethical integrity is not only vital for their well‐being but also essential for improving overall maternal healthcare outcomes. Addressing these issues requires institutional reforms, improved support systems, and trauma‐informed approaches that prioritize healthcare workers, women, birthing people and families.

**FIGURE 6:**
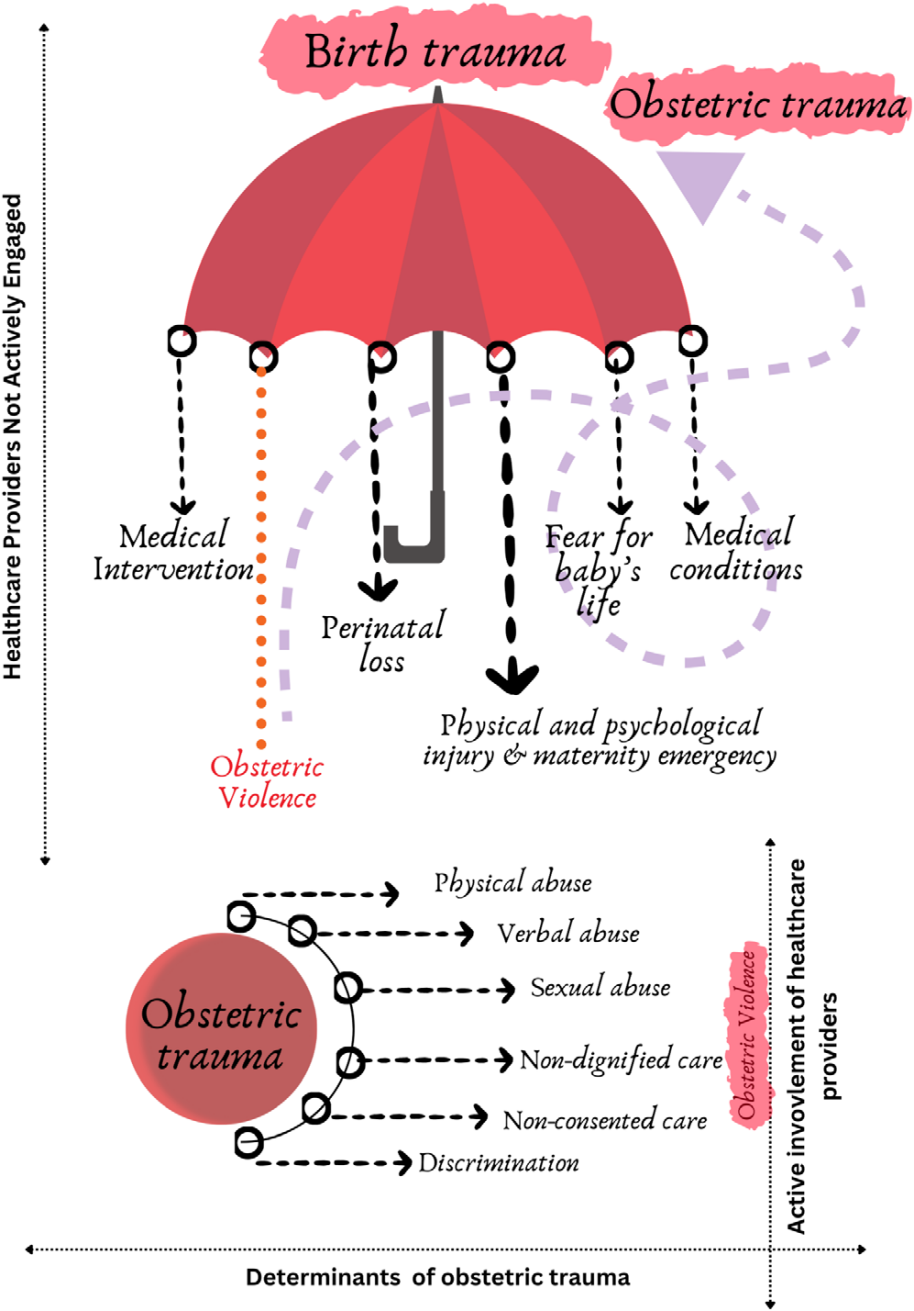
Introducing “Obstetric Trauma” to highlight a critical aspect of childbirth experience. [Colour figure can be viewed at wileyonlinelibrary.com]

Considering the critical gaps and conceptual confusion between the subjective trauma arising from childbirth experiences (birth trauma) and the trauma specifically resulting from abuse, coercion, and neglect by healthcare providers (obstetric violence), we are suggesting a new term, “obstetric trauma” A graphical presentation has been provided to illustrate the concept (Figure 6). Obstetric trauma would specifically indicate the consequences of obstetric violence resulting from mistreatment and coercion. This term would not only help distinguish it from birth trauma but also emphasize the systemic and avoidable nature of trauma. By framing obstetric trauma as a distinct concept, we can focus on the structural and institutional factors that perpetuate obstetric violence and its impact on individuals. It would also help guide targeted interventions, policy changes, and support systems aimed at preventing obstetric violence and addressing its effects, ultimately promoting more respectful and equitable maternity care. Even if the impact of obstetric violence is similar to the impact of birth trauma, using the new term will specifically acknowledge the direct link between trauma and harmful, avoidable practices perpetrated by healthcare providers. Using the term obstetric trauma may improve recognition, reporting, and interventions targeting obstetric violence. Training programs should inform the promotion of respectful maternity care among healthcare providers, which can minimize the act of obstetric violence. Muting the act of obstetric violence and not recognizing and acknowledging it is deeply problematic. Healthcare providers should develop knowledge on trauma‐informed care practices to support birthing persons, essential for eliminating subjective traumatic birthing experiences.

## 5 Conclusion

This concept analysis explains the intersection and distinctions between the two concepts obstetric violence and birth trauma. Both concepts have distinct antecedents and attributes that lead to similar consequences for birthing people. Conceptual clarity will improve as researchers delineate the overlap by using terms like obstetric trauma to describe obstetric violence‐driven traumatic births. The new terminology will help to sharpen the language used in advocacy, data collection, and healthcare guidelines, and ultimately the implementation of respectful maternity care.

## Ethics Statement

Ethical approval was not required, as the study was a concept analysis and did not involve participants.

## Conflicts of Interest

The authors declare no conflicts of interest.

## Supporting information


**Data S1:** Supporting Information.

**Data S2:** Supporting Information.

## Data Availability

The data that support the findings of this study are available on request from the corresponding author. The data are not publicly available due to privacy or ethical restrictions.
