# Supplementary material for: A Simultaneous Concept Analysis to Provide Clarity Between Obstetric Violence and Birth Trauma
Source: Birth. 2025 Sep 12;53(2):276–88. doi: 10.1111/birt.70019 (PMC13156660; doi:10.1111/birt.70019)
Supplement: Supplementary file 1 — Data S1: Supporting Information. [file BIRT-53-276-s002.docx]

| Obstetric violence | “the appropriation of the body and reproductive processes of women by health personnel, which is expressed as dehumanised treatment, an abuse of medication, and to convert the natural processes into pathological ones, bringing with it loss of autonomy and the ability to decide freely about their bodies and sexuality, negatively impacting the quality of life of women.” (Pérez D’gregorio, 2010, p. 201) |
| --- | --- |
|  | “an assemblage of disciplinary, bodily and material relations that are shaped by racialised, medicalised and classes norms about good patients, good women and good birthing bodies.” (Chadwick, 2017, p. 504) |
| Disrespect and abuse during childbirth | “the individual disrespect and abuse (i.e. specific provider behaviour experienced or intended as disrespectful or humiliating such as slapping or scolding women) and the structural disrespect and abuse (i.e. systemic deficiencies that create a disrespectful and abusive environment, such as overcrowded and understaffed maternity ward where women deliver on the floor, alone, in unhygienic conditions).”  (Freedman et al., 2014, p. 915) |
| Slow violence | “…a violence that occurs gradually and out of sight; a delayed destruction often dispersed across time and space.” (Nixon, 2011, p. 2) |
| Gentle violence | It occurs in care environments where women become submissive, compliant body subjects who willingly accept their role as patient (specifically in obstetric practices). (Chadwick, 2018) |
| Structural  violence | “social forces that create and maintain inequalities within and between social groups, which make way for conditions where interpersonal maltreatment and violence may be enacted… the essence of structural violence lies in the indirect, systematic and often invisible infliction of harm on individuals by social forces that disable individual from having their basic needs met.” (Miltenburg et al., 2018, p.2) |

Table 1: A detailed description of the definitions of obstetric violence.
