# Supplementary material for: A Simultaneous Concept Analysis to Provide Clarity Between Obstetric Violence and Birth Trauma
Source: Birth. 2025 Sep 12;53(2):276–88. doi: 10.1111/birt.70019 (PMC13156660; doi:10.1111/birt.70019)
Supplement: Supplementary file 2 — Data S2: Supporting Information. [file BIRT-53-276-s001.docx]

**Obstetric violence included study:**

Abuya, T., Ndwiga, C., Ritter, J., Kanya, L., Bellows, B., Binkin, N., & Warren, C. E. (2015). The effect of a multi-component intervention on disrespect and abuse during childbirth in Kenya. BMC Pregnancy and Childbirth, 15(1). https://doi.org/10.1186/s12884-015-0645-6

Adinew, Y. M., Kelly, J., Smith, M., & Marshall, A. (2021). Women’s perspectives on disrespect and abuse during facility-based childbirth in Ethiopia: a qualitative study. BMC Pregnancy and Childbirth, 13, 1181–1195. https://doi.org/10.1186/s12884-023-05762-8

Afulani, P. A., Kelly, A. M., Buback, L., Asunka, J., Kirumbi, L., & Lyndon, A. (2020). Providers’ perceptions of disrespect and abuse during childbirth: A mixed-methods study in Kenya. Health Policy and Planning, 35(5), 577–586. https://doi.org/10.1093/heapol/czaa009

Ahmed, H. M. (2022). Rate and Types of Childbirth Mistreatment and Abuse and Its Association with Satisfaction with Birth Care: A Cross-Sectional Study of 1196 Kurdish Women. Maternal and Child Health Journal, 26(6), 1322–1327. https://doi.org/10.1007/s10995-021-03318-w

Ajayi, A. I., Gebrekristos, L. T., Otukpa, E., & Kabiru, C. W. (2023). Adolescents’ experience of mistreatment and abuse during childbirth: A cross-sectional community survey in a low-income informal settlement in Nairobi, Kenya. BMJ Global Health, 8(11), 1–8. https://doi.org/10.1136/bmjgh-2023-013268

Aşci, Ö., & Bal, M. D. (2023). The prevalence of obstetric violence experienced by women during childbirth care and its associated factors in Türkiye: A cross-sectional study. Midwifery, 124. https://doi.org/10.1016/j.midw.2023.103766

Asefa, A., Bekele, D., Morgan, A., & Kermode, M. (2018). Service providers’ experiences of disrespectful and abusive behavior towards women during facility based childbirth in Addis Ababa, Ethiopia. Reproductive Health, 15(1), 4. https://doi.org/10.1186/s12978-017-0449-4

Avcı, N., & Kaydırak, M. M. (2023). A qualitative study of women’s experiences with obstetric violence during childbirth in Turkey. Midwifery, 121. https://doi.org/10.1016/j.midw.2023.103658

Azhar, Z., Oyebode, O., & Masud, H. (2018). Disrespect and abuse during childbirth in district Gujrat , Pakistan : A quest for respectful maternity care. 1–11.

Azzam, O. A., Sindiani, A. M., Eyalsalman, M. M., Odeh, M. K., AbedAlkareem, K. Y., Albanna, S. A., Abdulrahman, E. M., Abukhadrah, W. Q., Hazaimeh, H. O., Zaghloul, A. A., & Mahgoub, S. S. (2023). Obstetric Violence among Pregnant Jordanian Women: An Observational Study between the Private and Public Hospitals in Jordan. Healthcare (Switzerland), 11(5), 1–10. https://doi.org/10.3390/healthcare11050654

Balde, M. D., Bangoura, A., Diallo, B. A., Sall, O., Balde, H., Niakate, A. S., Vogel, J. P., & Bohren, M. A. (2017). A qualitative study of women’s and health providers’ attitudes and acceptability of mistreatment during childbirth in health facilities in Guinea. Reproductive Health, 14(1), 1–13. https://doi.org/10.1186/s12978-016-0262-5

Bekele, W., Bayou, N. B., & Garedew, M. G. (2020). Magnitude of disrespectful and abusive care among women during facility-based childbirth in Shambu town, Horro Guduru Wollega zone, Ethiopia,. Midwifery, 83, 102629. https://doi.org/10.1016/j.midw.2020.102629

Bhattacharya, S., & Sundari Ravindran, T. K. (2018). Silent voices: Institutional disrespect and abuse during delivery among women of Varanasi district, northern India. BMC Pregnancy and Childbirth, 18(1), 1–8. https://doi.org/10.1186/s12884-018-1970-3

Biresaw, W., & Shegaw, Z. (2018). Compassionate and respectful maternity care during facility based child birth and women’s intent to use maternity service in Bahir Dar, Ethiopia. BMC Pregnancy and Childbirth, 18(1), 1–9. chrome-extension://efaidnbmnnnibpcajpcglclefindmkaj/https://www.ncbi.nlm.nih.gov/pmc/articles/PMC6038196/pdf/12884_2018_Article_1909.pdf

Bobo, F. T., Kasaye, H. K., Etana, B., Woldie, M., & Feyissa, T. R. (2019). Disrespect and abuse during childbirth in Western Ethiopia: Should women continue to tolerate? PLoS ONE, 14(6), 1–15. https://doi.org/10.1371/journal.pone.0217126

Bohren, M. A., Mehrtash, H., Fawole, B., Maung, T. M., Balde, M. D., Maya, E., Thwin, S. S., Aderoba, A. K., Vogel, J. P., Irinyenikan, T. A., Adeyanju, A. O., Mon, N. O., Adu-Bonsaffoh, K., Landoulsi, S., Guure, C., Adanu, R., Diallo, B. A., Gülmezoglu, A. M., Soumah, A. M., … Tunçalp, Ö. (2019). How women are treated during facility-based childbirth in four countries: a cross-sectional study with labour observations and community-based surveys. The Lancet, 394(10210), 1750–1763. https://doi.org/10.1016/S0140-6736(19)31992-0

Bohren, M. A., Vogel, J. P., Hunter, E. C., Lutsiv, O., Makh, S. K., Souza, J. P., Aguiar, C., Saraiva Coneglian, F., Diniz, A. L. A., Tunçalp, Ö., Javadi, D., Oladapo, O. T., Khosla, R., Hindin, M. J., & Gülmezoglu, A. M. (2015). The Mistreatment of Women during Childbirth in Health Facilities Globally: A Mixed-Methods Systematic Review. PLoS Medicine, 12(6), 1–32. https://doi.org/10.1371/journal.pmed.1001847

Bradley, S., McCourt, C., Rayment, J., & Parmar, D. (2019). Midwives’ perspectives on (dis)respectful intrapartum care during facility-based delivery in sub-Saharan Africa: A qualitative systematic review and meta-synthesis. Reproductive Health, 16(1), 1–16. https://doi.org/10.1186/s12978-019-0773-y

Chadwick, R. (2017). Ambiguous subjects: Obstetric violence, assemblage and South African birth narratives. Feminism and Psychology, 27(4), 489–509. https://doi.org/10.1177/0959353517692607

da Conceição, H. N., Gonçalves, C. F. G., Mascarenhas, M. D. M., Rodrigues, M. T. P., Madeiro, A. P., & Conceição, H. N. (2023). Disrespect and abuse during childbirth and postpartum depression: a scoping review. Cadernos de Saude Publica, 39(5), 1–13. https://doi.org/10.1590/0102-311XEN236922

Dey, A., Shakya, H. B., Chandurkar, D., Kumar, S., Das, A. K., Anthony, J., Shetye, M., Krishnan, S., Silverman, J. G., & Raj, A. (2017). Discordance in self-report and observation data on mistreatment of women by providers during childbirth in Uttar Pradesh. Reproductive Health, 14(1), 1–13. https://doi.org/10.1186/s12978-017-0409-z

Dwekat, I. M. M., Tengku Ismail, T. A., Ibrahim, M. I., & Ghrayeb, F. (2021). Exploring factors contributing to mistreatment of women during childbirth in West Bank, Palestine. Women and Birth, 34(4), 344–351. https://doi.org/10.1016/j.wombi.2020.07.004

Ezeanochie, M. C., & Yamah, O. E. (2023). Prevalence and factors associated with disrespect and abuse among women who delivered in a University Teaching Hospital in Nigeria. International Journal of Gynecology and Obstetrics, 161(3), 685–691. https://doi.org/10.1002/ijgo.14812

Faheem, A. (2021). The nature of obstetric violence and the organisational context of its manifestation in India: a systematic review. Sexual and Reproductive Health Matters, 29(2), 427–435. https://doi.org/10.1080/26410397.2021.2004634

Fors, M., Falcón, K., Brandao, T., López, M., & Mena-Tudela, D. (2024). Investigating Obstetric Violence in Ecuador: A Cross-Sectional Study Spanning the Last Several Years. Healthcare (Switzerland), 12(15), 1–12. https://doi.org/10.3390/healthcare12151480

Galle, A., Manaharlal, H., Cumbane, E., Picardo, J., Griffin, S., Osman, N., Roelens, K., & Degomme, O. (2019). Disrespect and abuse during facility-based childbirth in southern Mozambique: a cross-sectional study. BMC Pregnancy and Childbirth, 19(1), 1–13. https://doi.org/10.1186/s12884-019-2532-z

Garcia, L. M. (2020). A concept analysis of obstetric violence in the United States of America. Nursing Forum, 55(4), 654–663. https://doi.org/10.1111/nuf.12482

Ghimire, N. P., Joshi, S. K., Dahal, P., & Swahnberg, K. (2021). Women’s experience of disrespect and abuse during institutional delivery in biratnagar, nepal. International Journal of Environmental Research and Public Health, 18(18). https://doi.org/10.3390/ijerph18189612

Goli, S., Ganguly, D., Chakravorty, S., Siddiqui, M. Z., Ram, H., Rammohan, A., & Acharya, S. S. (2019). Labour room violence in Uttar Pradesh, India: Evidence from longitudinal study of pregnancy and childbirth. BMJ Open, 9(7), 1–6. https://doi.org/10.1136/bmjopen-2018-028688

Gurung, R., & Bask, M. (2024). Does mistreatment during institutional childbirth increase the likelihood of experiencing postpartum depressive symptoms? A prospective cohort study in Nepal. Global Health Action, 17(1), 1–13. https://doi.org/10.1080/16549716.2024.2381312

Gurung, R., Moinuddin, M., Sunny, A. K., Bhandari, A., Axelin, A., & Kc, A. (2022). Mistreatment during childbirth and postnatal period reported by women in Nepal —a multicentric prevalence study. BMC Pregnancy and Childbirth, 22(1), 1–10. https://doi.org/10.1186/s12884-022-04639-6

Guure, C., Aviisah, P. A., Adu-Bonsaffoh, K., Mehrtash, H., Aderoba, A. K., Irinyenikan, T. A., Balde, M. D., Adeyanju, O., Maung, T. M., Tunçalp, Ö., & Maya, E. (2023). Mistreatment of women during childbirth and postpartum depression: secondary analysis of WHO community survey across four countries. BMJ Global Health, 8(8), 1–11. https://doi.org/10.1136/bmjgh-2023-011705

Hajizadeh, K., Vaezi, M., Meedya, S., Mohammad Alizadeh Charandabi, S., & Mirghafourvand, M. (2023). Iranian mother’s perspectives about aspects and determinants of disrespect and abuse during labor and delivery: a qualitative study. Women and Health, 63(8), 623–636. https://doi.org/10.1080/03630242.2023.2250466

Hameed, W., Uddin, M., & Avan, B. I. (2021). Are underprivileged and less empowered women deprived of respectful maternity care: Inequities in childbirth experiences in public health facilities in Pakistan. PLoS ONE, 16(4 April), 1–17. https://doi.org/10.1371/journal.pone.0249874

Irinyenikan, T. A., Aderoba, A. K., Fawole, O., Adeyanju, O., Mehrtash, H., Adu-Bonsaffoh, K., Maung, T. M., Balde, M. D., Vogel, J. P., Plesons, M., Chandra-Mouli, V., Tunçalp, Ö., & Bohren, M. A. (2022). Adolescent experiences of mistreatment during childbirth in health facilities: Secondary analysis of a community-based survey in four countries. BMJ Global Health, 5, 1–11. https://doi.org/10.1136/bmjgh-2021-007954

Ishola, F., Owolabi, O., & Filippi, V. (2017). Disrespect and abuse of women during childbirth in Nigeria : A systematic review. PLoS ONE, 12(3), 1–17. https://doi.org/https://doi.org/10.1371/journal. pone.0174084

Jungari, S., Sharma, B., & Wagh, D. (2021). Beyond Maternal Mortality: A Systematic Review of Evidences on Mistreatment and Disrespect During Childbirth in Health Facilities in India. Trauma, Violence, and Abuse, 22(4), 739–751. https://doi.org/10.1177/1524838019881719

Kabakian-Khasholian, T., Makhoul, J., & Ghusayni, A. (2022). “A person who does not have money does not enter”: a qualitative study on refugee women’s experiences of respectful maternity care. BMC Pregnancy and Childbirth, 22(1), 1–10. https://doi.org/10.1186/s12884-022-05083-2

Kassa, Z. Y., & Husen, S. (2019). Disrespectful and abusive behavior during childbirth and maternity care in Ethiopia: A systematic review and meta-analysis. BMC Research Notes, 12(1), 2–7. https://doi.org/10.1186/s13104-019-4118-2

Kassa, Z. Y., Tsegaye, B., & Abeje, A. (2020). Disrespect and abuse of women during the process of childbirth at health facilities in sub-Saharan Africa: A systematic review and meta-analysis. BMC International Health and Human Rights, 20(1), 1–9. https://doi.org/10.1186/s12914-020-00242-y

Keedle, H., Keedle, W., & Dahlen, H. G. (2024). Dehumanized, Violated, and Powerless: An Australian Survey of Women’s Experiences of Obstetric Violence in the Past 5 Years. Violence Against Women, 30(9), 2320–2344. https://doi.org/10.1177/10778012221140138

Kruk, M. E., Kujawski, S., Mbaruku, G., Ramsey, K., Moyo, W., & Freedman, L. P. (2018). Disrespectful and abusive treatment during facility delivery in Tanzania: A facility and community survey. Health Policy and Planning, 33(1), e26–e33. https://doi.org/10.1093/heapol/czu079

Lansky, S., De Souza, K. V., De Morais Peixoto, E. R., Oliveira, B. J., Diniz, C. S. G., Vieira, N. F., De Oliveira Cunha, R., & De Lima Friche, A. A. (2019). Obstetric violence: influences of the senses of birth exhibition in pregnant women childbirth experience. Ciencia e Saude Coletiva, 24(8), 2811–2824. https://doi.org/10.1590/1413-81232018248.30102017

Leavy, E., Cortet, M., Huissoud, C., Desplanches, T., Sormani, J., Viaux-Savelon, S., Dupont, C., Pichon, S., & Gaucher, L. (2023). Disrespect during childbirth and postpartum mental health: a French cohort study. BMC Pregnancy and Childbirth, 23(1), 4–11. https://doi.org/10.1186/s12884-023-05551-3

Leijerzapf, D. R., van der Pijl, M. S. G., Hollander, M. H., Kingma, E., de Jonge, A., & Verhoeven, C. J. M. (2024). Experienced disrespect & abuse during childbirth and associated birth characteristics: a cross-sectional survey in the Netherlands. BMC Pregnancy and Childbirth, 24(1), 1–15. https://doi.org/10.1186/s12884-024-06360-y

Leite, T. H., Carvalho, T. D. G., Marques, E. S., Pereira, A. P. E., da Silva, A. A. M., Nakamura-Pereira, M., & Leal, M. do C. (2021). The association between mistreatment of women during childbirth and postnatal maternal and child health care: Findings from “Birth in Brazil.” Women and Birth, 35(1), e28–e40. https://doi.org/10.1016/j.wombi.2021.02.006

Leite, T. H., Carvalho, T. D. G., Marques, E. S., Pereira, A. P. E., da Silva, A. A. M., Nakamura-Pereira, M., & Leal, M. do C. (2022). The association between mistreatment of women during childbirth and postnatal maternal and child health care: Findings from “Birth in Brazil.” Women and Birth, 35(1), e28–e40. https://doi.org/10.1016/j.wombi.2021.02.006

Leite, T. H., Marques, E. S., Corrêa, R. G., Leal, M. do C., Olegário, B. da C. D., Costa, R. M. da, & Mesenburg, M. A. (2024). Epidemiology of obstetric violence: a narrative review of the Brazilian context. Ciência & Saúde Coletiva, 29(9). https://doi.org/10.1590/1413-81232024299.12222023en

Leite, T. H., Pereira, A. P. E., Leal, M. do C., & da Silva, A. A. M. (2020). Disrespect and abuse towards women during childbirth and postpartum depression: findings from Birth in Brazil Study. Journal of Affective Disorders, 273(April), 391–401. https://doi.org/10.1016/j.jad.2020.04.052

Madeiro, A., Rufino, A. C., Acaqui, R. F., Barbosa, C. M., Martins, V. M. M. L., & de Sousa, A. M. C. (2022). Disrespect and abuse during childbirth in maternity hospitals in Piauí, Brazil: A cross-sectional study. International Journal of Gynecology and Obstetrics, 159(3), 961–967. https://doi.org/10.1002/ijgo.14317

Madhiwalla, N., Ghoshal, R., Mavani, P., & Roy, N. (2018). Identifying disrespect and abuse in organisational culture: a study of two hospitals in Mumbai, India. Reproductive Health Matters, 0(0), 1–12. https://doi.org/10.1080/09688080.2018.1502021

Malatji, R., & Madiba, S. (2020). Disrespect and abuse experienced by women during childbirth in midwife-led obstetric units in Tshwane district, South Africa: A qualitative study. International Journal of Environmental Research and Public Health, 17(10), 12–14. https://doi.org/10.3390/ijerph17103667

Maldie, M., Egata, G., Chanie, M. G., Muche, A., Dewau, R., Worku, N., Alemu, M. D., Ewunetie, G. E., Birhane, T., Addisu, E., Ayele, W. M., & Adane, M. (2021). Magnitude and associated factors of disrespect and abusive care among laboring mothers at public health facilities in Borena District, South Wollo, Ethiopia. PLoS ONE, 16(11 November), 1–18. https://doi.org/10.1371/journal.pone.0256951

Martínez-Galiano, J. M., Martinez-Vazquez, S., Rodríguez-Almagro, J., & Hernández-Martinez, A. (2021). The magnitude of the problem of obstetric violence and its associated factors: A cross-sectional study. Women and Birth, 34(5), e526–e536. https://doi.org/10.1016/j.wombi.2020.10.002

Martinez-Vázquez, S., Rodríguez-Almagro, J., Hernández-Martínez, A., Delgado-Rodríguez, M., & Martínez-Galiano, J. M. (2021). Obstetric factors associated with postpartum post-traumatic stress disorder after spontaneous vaginal birth. Birth, 48(3), 406–415. https://doi.org/10.1111/birt.12550

Martinez-Vázquez, S., Rodríguez-Almagro, J., Hernández-Martínez, A., & Martínez-Galiano, J. M. (2021). Factors associated with postpartum post-traumatic stress disorder (Ptsd) following obstetric violence: A cross-sectional study. Journal of Personalized Medicine, 11(5). https://doi.org/10.3390/jpm11050338

Maung, T. M., Show, K. L., Mon, N. O., Tunçalp, Ö., Aye, N. S., Soe, Y. Y., & Bohren, M. A. (2020). A qualitative study on acceptability of the mistreatment of women during childbirth in Myanmar. Reproductive Health, 17(1), 1–14. https://doi.org/10.1186/s12978-020-0907-2

Maya, E. T., Adu-Bonsaffoh, K., Dako-Gyeke, P., Badzi, C., Vogel, J. P., Bohren, M. A., & Adanu, R. (2018). Women’s perspectives of mistreatment during childbirth at health facilities in Ghana: findings from a qualitative study. Reproductive Health Matters, 26(53), 70–87. https://doi.org/10.1080/09688080.2018.1502020

Mayra, K., Matthews, Z., & Padmadas, S. S. (2022). Why do some health care providers disrespect and abuse women during childbirth in India? Women and Birth, 35(1), e49–e59. https://doi.org/10.1016/j.wombi.2021.02.003

Mayra, K., Sandall, J., Matthews, Z., & Padmadas, S. S. (2022). Breaking the silence about obstetric violence: Body mapping women’s narratives of respect, disrespect and abuse during childbirth in Bihar, India. BMC Pregnancy and Childbirth, 22(1), 318. https://doi.org/10.1186/s12884-022-04503-7

Mena-Tudela, D., Iglesias-Casás, S., González-Chordá, V. M., Cervera-Gasch, Á., Andreu-Pejó, L., & Valero-Chilleron, M. J. (2020). Obstetric violence in Spain (Part I): Women’s perception and interterritorial differences. International Journal of Environmental Research and Public Health, 17(21), 1–14. https://doi.org/10.3390/ijerph17217726

Mengesha, M. B., Desta, A. G., Maeruf, H., & Hidru, H. D. (2020). Disrespect and abuse during childbirth in Ethiopia: A systematic review. BioMed Research International, 2020, 1–14. https://doi.org/10.1155/2020/8186070

Mesenburg, M. A., Victora, C. G., Jacob Serruya, S., Ponce De León, R., Damaso, A. H., Domingues, M. R., & Da Silveira, M. F. (2018). Disrespect and abuse of women during the process of childbirth in the 2015 Pelotas birth cohort Prof. Suellen Miller. Reproductive Health, 15(1), 1–8. https://doi.org/10.1186/s12978-018-0495-6

Meyer, S., Cignacco, E., Monteverde, S., Trachsel, M., Raio, L., & Oelhafen, S. (2022). “We felt like part of a production system”: A qualitative study on women’s experiences of mistreatment during childbirth in Switzerland. PLoS ONE, 17(2 February), 1–21. https://doi.org/10.1371/journal.pone.0264119

Mihret, M. S. (2019). Obstetric violence and its associated factors among postnatal women in a Specialized Comprehensive Hospital, Amhara Region, Northwest Ethiopia. BMC Research Notes, 12(1), 1–7. https://doi.org/10.1186/s13104-019-4614-4

Miltenburg, A. S., van Pelt, S., Meguid, T., & Sundby, J. (2018). Disrespect and abuse in maternity care: individual consequences of structural violence. Reproductive Health Matters, 26(53), 88–106. https://doi.org/10.1080/09688080.2018.1502023

Mirzania, M., Shakibazadeh, E., Bohren, M. A., Hantoushzadeh, S., Babaey, F., Khajavi, A., & Foroushani, A. R. (2023). Mistreatment of women during childbirth and its influencing factors in public maternity hospitals in Tehran, Iran: a multi-stakeholder qualitative study. Reproductive Health, 20(1), 1–14. https://doi.org/10.1186/s12978-023-01620-0

Molla, W., Wudneh, A., & Tilahun, R. (2022). Obstetric violence and associated factors among women during facility based childbirth at Gedeo Zone, South Ethiopia. BMC Pregnancy and Childbirth, 22(1), 1–14. https://doi.org/10.1186/s12884-022-04895-6

Montesinos-Segura, R., Urrunaga-Pastor, D., Mendoza-Chuctaya, G., Taype-Rondan, A., Helguero-Santin, L. M., Martinez-Ninanqui, F. W., Centeno, D. L., Jiménez-Meza, Y., Taminche-Canayo, R. C., Paucar-Tito, L., & Villamonte-Calanche, W. (2018). Disrespect and abuse during childbirth in fourteen hospitals in nine cities of Peru. International Journal of Gynecology and Obstetrics, 140(2), 184–190. https://doi.org/10.1002/ijgo.12353

Mselle, L. T., Kohi, T. W., & Dol, J. (2019). Humanizing birth in Tanzania: A qualitative study on the (mis) treatment of women during childbirth from the perspective of mothers and fathers. BMC Pregnancy and Childbirth, 19(1), 1–11. https://doi.org/10.1186/s12884-019-2385-5

Okedo-Alex, I. N., Akamike, I. C., Igwilo, U., & Onwasigwe, C. N. (2021). What factors are associated with forms of mistreatment during facility-based childbirth? A survey of referral health facilities in south-east Nigeria. Journal of Biosocial Science, 776–791. https://doi.org/10.1017/S002193202100047X

Oluoch-Aridi, J., Smith-Oka, V., Milan, E., & Dowd, R. (2018). Exploring mistreatment of women during childbirth in a peri-urban setting in Kenya: Experiences and perceptions of women and healthcare providers. Reproductive Health, 15(1), 1–14. https://doi.org/10.1186/s12978-018-0643-z

Paiz, J. C., de Jezus Castro, S. M., Giugliani, E. R. J., dos Santos Ahne, S. M., Aqua, C. B. D., & Giugliani, C. (2022). Association between mistreatment of women during childbirth and symptoms suggestive of postpartum depression. BMC Pregnancy and Childbirth, 22(1), 1–10. https://doi.org/10.1186/s12884-022-04978-4

Pazandeh, F., Moridi, M., & Safari, K. (2023). Labouring women perspectives on mistreatment during childbirth: a qualitative study. Nursing Ethics, 0(0), 1–13. https://doi.org/10.1177/09697330231158732

Perera, D., Lund, R., Swahnberg, K., Schei, B., Infanti, J. J., Darj, E., Lukasse, M., Bjørngaard, J. H., Joshi, S. K., Rishal, P., Koju, R., Pun, K. D., Wijewardena, K., Muzrif, M. M., & Campbell, J. C. (2018). “When helpers hurt”: Women’s and midwives’ stories of obstetric violence in state health institutions, Colombo district, Sri Lanka. BMC Pregnancy and Childbirth, 18(1), 1–12. https://doi.org/10.1186/s12884-018-1869-z

Pérez D’gregorio, R. (2010). Obstetric violence: A new legal term introduced in Venezuela. International Journal of Gynecology and Obstetrics, 111(3), 201–202. https://doi.org/10.1016/j.ijgo.2010.09.002

Ramírez-Perdomo, C. A., Flórez-González, A. M., & Mateus-Peña, J. D. (2024). Women’s Experiences in the Childbirth Care Process: An Integrative Review. Aquichan, 24(2). https://doi.org/10.5294/aqui.2024.24.2.5

Sadler, M., Santos, M. J., Ruiz-Berdún, D., Rojas, G. L., Skoko, E., Gillen, P., & Clausen, J. A. (2016). Moving beyond disrespect and abuse: addressing the structural dimensions of obstetric violence. Reproductive Health Matters, 24(47), 47–55. https://doi.org/10.1016/j.rhm.2016.04.002

Santiago, R. V., Monreal, L. A., Rojas Carmona, A., & Domínguez, M. S. (2018). “If we’re here, it’s only because we have no money.” discrimination and violence in Mexican maternity wards. BMC Pregnancy and Childbirth, 18(1), 1–10. https://doi.org/10.1186/s12884-018-1897-8

Scandurra, C., Zapparella, R., Policastro, M., Continisio, G. I., Ammendola, A., Bochicchio, V., Maldonato, N. M., & Locci, M. (2022). Obstetric violence in a group of Italian women: socio-demographic predictors and effects on mental health. Culture, Health and Sexuality, 24(11), 1466–1480. https://doi.org/10.1080/13691058.2021.1970812

Scotland, M. (2020). Birth Shock. Pinter and Martin.

Sen, G., Reddy, B., & Iyer, A. (2018). Beyond measurement: the drivers of disrespect and abuse in obstetric care. Reproductive Health Matters, 26(53), 6–18. https://doi.org/10.1080/09688080.2018.1508173

Sharma, G., Penn-Kekana, L., Halder, K., & Filippi, V. (2019). An investigation into mistreatment of women during labour and childbirth in maternity care facilities in Uttar Pradesh, India: A mixed methods study. Reproductive Health, 16(1), 1–16. https://doi.org/10.1186/s12978-019-0668-y

Sheferaw, E. D., Kim, Y. M., Van Den Akker, T., & Stekelenburg, J. (2019). Mistreatment of women in public health facilities of Ethiopia. Reproductive Health, 16(1), 1–10. https://doi.org/10.1186/s12978-019-0781-y

Shrivastava, S., & Sivakami, M. (2020). Evidence of “obstetric violence” in India: An integrative review. Journal of Biosocial Science, 52(4), 610–628. https://doi.org/10.1017/S0021932019000695

Silva-Fernandez, C. S., de la Calle, M., Arribas, S. M., Garrosa, E., & Ramiro-Cortijo, D. (2023). Factors Associated with Obstetric Violence Implicated in the Development of Postpartum Depression and Post-Traumatic Stress Disorder: A Systematic Review. Nursing Reports, 13(4), 1553–1576. https://doi.org/10.3390/nursrep13040130

Silveira, M. F., Mesenburg, M. A., Bertoldi, A. D., De Mola, C. L., Bassani, D. G., Domingues, M. R., Stein, A., & Coll, C. V. N. (2019). The association between disrespect and abuse of women during childbirth and postpartum depression: Findings from the 2015 Pelotas birth cohort study. Journal of Affective Disorders, 256(January), 441–447. https://doi.org/10.1016/j.jad.2019.06.016

Siraj, A., Teka, W., & Hebo, H. (2019). Prevalence of disrespect and abuse during facility based child birth and associated factors, Jimma University Medical Center, Southwest Ethiopia. BMC Pregnancy and Childbirth, 19(1), 1–9. https://doi.org/10.1186/s12884-019-2332-5

Sudhinaraset, M., Treleaven, E., Melo, J., Singh, K., & Diamond-Smith, N. (2016). Women’s status and experiences of mistreatment during childbirth in Uttar Pradesh: A mixed methods study using cultural health capital theory. BMC Pregnancy and Childbirth, 16(1). https://doi.org/10.1186/s12884-016-1124-4

Taghizadeh, Z., Ebadi, A., & Jaafarpour, M. (2021). Childbirth violence-based negative health consequences: a qualitative study in Iranian women. BMC Pregnancy and Childbirth, 21(1), 1–10. https://doi.org/10.1186/s12884-021-03986-0

Ukke, G. G., Gurara, M. K., & Boynito, W. G. (2019). Disrespect and abuse of women during childbirth in public health facilities in Arba Minch town, south Ethiopia – a cross-sectional study. PLoS ONE, 14(4), 1–17. https://doi.org/10.1371/journal.pone.0205545

Vedam, S., Stoll, K., Taiwo, T. K., Rubashkin, N., Cheyney, M., Strauss, N., McLemore, M., Cadena, M., Nethery, E., Rushton, E., Schummers, L., & Declercq, E. (2019). The Giving Voice to Mothers study: Inequity and mistreatment during pregnancy and childbirth in the United States. Reproductive Health, 16(1), 1–18. https://doi.org/10.1186/s12978-019-0729-2

Wahdan, Y., & Abu-Rmeileh, N. M. E. (2023). The association between labor companionship and obstetric violence during childbirth in health facilities in five facilities in the occupied Palestinian territory. BMC Pregnancy and Childbirth, 23(1), 1–8. https://doi.org/10.1186/s12884-023-05811-2

Warren, C. E., Njue, R., Ndwiga, C., & Abuya, T. (2017). Manifestations and drivers of mistreatment of women during childbirth in Kenya: Implications for measurement and developing interventions. BMC Pregnancy and Childbirth, 17(1), 1–14. https://doi.org/10.1186/s12884-017-1288-6

Werdofa, H. M., Thoresen, L., Lulseged, B., & Lindahl, A. K. (2023). ‘I believe respect means providing necessary treatment on time’ - a qualitative study of health care providers’ perspectives on disrespect and abuse during childbirth in Southwest Ethiopia. BMC Pregnancy and Childbirth, 23(1), 1–11. https://doi.org/10.1186/s12884-023-05567-9

Yalley, A. A., Abioye, D., Appiah, S. C. Y., & Hoeffler, A. (2023). Abuse and humiliation in the delivery room: Prevalence and associated factors of obstetric violence in Ghana. Frontiers in Public Health, 11(1). https://doi.org/10.3389/fpubh.2023.988961

Yohannes, E., Moti, G., Gelan, G., Creedy, D. K., Gabriel, L., & Hastie, C. (2024). Impact of disrespectful maternity care on childbirth complications: a multicentre cross-sectional study in Ethiopia. BMC Pregnancy and Childbirth, 24(1), 1–9. https://doi.org/10.1186/s12884-024-06574-0

Yupanqui-Concha, A., Hichins-Arismendi, M., Mandiola-Godoy, D., Rodríguez-Garrido, P., & Rotarou, E. S. (2024). Accessing Sexual and Reproductive Health Services in Chile: Women with Disabilities and Their Experience with Gynaecological and Obstetric Violence. Sexuality Research and Social Policy, 21(2), 690–703. https://doi.org/10.1007/s13178-024-00942-3

**Birth trauma included study:**

Abdollahpour, S., & Motaghi, Z. (2019). Lived Traumatic Childbirth Experiences of Newly Delivered Mothers Admitted to the Postpartum Ward: a Phenomenological Study. Journal of Caring Sciences, 8(1), 23–31. https://doi.org/10.15171/jcs.2019.004

Adewuya, A. O., Ologun, Y. A., & Ibigbami, O. S. (2006). Post-traumatic stress disorder after childbirth in Nigerian women: Prevalence and risk factors. BJOG: An International Journal of Obstetrics and Gynaecology, 113(3), 284–288. https://doi.org/10.1111/j.1471-0528.2006.00861.x

Aksu, D. F., & Serçekuş, P. (2023). Traumatic childbirth experiences, effects and coping: A qualitative study. Sexual and Reproductive Healthcare, 37(August), 1–5. https://doi.org/10.1016/j.srhc.2023.100898

Anderson, C. A. (2017). The trauma of birth. Health Care for Women International, 38(10), 999–1010. https://doi.org/10.1080/07399332.2017.1363208

Anderson, C., & Mccarley, M. (2013). Psychological Birth Trauma in Adolescents Experiencing an Early Birth. The Birth Trauma Association, 38(3), 171–176.

Anderson, C., & Perez, C. (2015). Adolescent psychological birth trauma following cesarean birth. Pediatric Nursing, 41(2), 78–83.

Ayers, S. (2017). Birth trauma and post-traumatic stress disorder: the importance of risk and resilience. Journal of Reproductive and Infant Psychology, 35(5), 427–430. https://doi.org/10.1080/02646838.2017.1386874

Bay, F., & Sayiner, F. D. (2021). Perception of traumatic childbirth of women and its relationship with postpartum depression. Women and Health, 61(5), 479–489. https://doi.org/10.1080/03630242.2021.1927287

Bayrı Bingöl, F. (2021). The Relationship Between Postpartum Maternal Traumatic Stress and Bonding. Journal of Psychiatric Nursing, 16(1654), 1–13. https://doi.org/10.14744/phd.2021.57704

Beck-Hiestermann, F. M. L., Hartung, L. K., Richert, N., Miethe, S., & Wiegand-Grefe, S. (2024). Are 6 more accurate than 4? The influence of different modes of delivery on postpartum depression and PTSD. BMC Pregnancy and Childbirth, 24(1), 1–11. https://doi.org/10.1186/s12884-024-06267-8

Beck, C. T. (2004). Birth trauma: In the eye of the beholder. Nursing Research, 53(1), 28–35. https://doi.org/10.1097/00006199-200401000-00005

Beck, C. T. (2015). Middle range theory of traumatic childbirth: The ever-widening ripple effect. Global Qualitative Nursing Research, 2015, 1–13. https://doi.org/10.1177/2333393615575313

Beck, C. T. (2017). The Anniversary of Birth Trauma: A Metaphor Analysis. The Journal of Perinatal Education, 26(4), 219–228. https://doi.org/10.1891/1058-1243.26.4.219

Beck, C. T. (2021). Subsequent Childbirth After a Previous Birth Trauma: A Metaphor Analysis. Issues in Mental Health Nursing, 42(10), 909–916. https://doi.org/10.1080/01612840.2021.1910759

Beck, C. T., & Watson, S. (2008). Impact of Birth Trauma on Breast-feeding: A Tale of two pathways. Nursing Research, 57(4), 228–236.

Beck, C. T., & Watson, S. (2010). Subsequent Childbirth After a Previous Traumatic Birth. Nursing Research, 59(4), 241–249.

Beck, C. T., & Watson, S. (2016). Posttraumatic Growth After Birth Trauma. MCN: The American Journal of Maternal/Child Nursing, 41(5), 264–271. https://doi.org/10.1097/NMC.0000000000000259

Berman, Z., Thiel, F., Kaimal, A. J., & Dekel, S. (2021). Association of sexual assault history with traumatic childbirth and subsequent PTSD. Archives of Women’s Mental Health, 24(5), 767–771. https://doi.org/10.1007/s00737-021-01129-0

Brown, A., Nielsen, J. D. J., Russo, K., Ayers, S., & Webb, R. (2022). The Journey towards resilience following a traumatic birth: A grounded theory. Midwifery, 104, 103204. https://doi.org/10.1016/j.midw.2021.103204

Çapik, A., & Durmaz, H. (2018). Fear of Childbirth, Postpartum Depression, and Birth-Related Variables as Predictors of Posttraumatic Stress Disorder After Childbirth. Worldviews on Evidence-Based Nursing, 15(6), 455–463. https://doi.org/10.1111/wvn.12326

Carter, J., Bick, D., Gallacher, D., & Chang, Y. S. (2022). Mode of birth and development of maternal postnatal post-traumatic stress disorder: A mixed-methods systematic review and meta-analysis. Birth, 49(4), 616–627. https://doi.org/10.1111/birt.12649

Chabbert, M., Panagiotou, D., & Wendland, J. (2021). Predictive factors of women’s subjective perception of childbirth experience: a systematic review of the literature. Journal of Reproductive and Infant Psychology, 39(1), 43–66. https://doi.org/10.1080/02646838.2020.1748582

Chen, Y., Ismail, F., Xiong, Z., Li, M., Chen, I., Wen, S. W., & Xie, R. hua. (2022). Association between perceived birth trauma and postpartum depression: A prospective cohort study in China. International Journal of Gynecology and Obstetrics, 157(3), 598–603. https://doi.org/10.1002/ijgo.13845

Cross, H., Krahé, C., Spiby, H., & Slade, P. (2023). Do antenatal preparation and obstetric complications and procedures interact to affect birth experience and postnatal mental health? BMC Pregnancy and Childbirth, 23(1), 1–14. https://doi.org/10.1186/s12884-023-05846-5

De Schepper, S., Vercauteren, T., Tersago, J., Jacquemyn, Y., Raes, F., & Franck, E. (2016). Post-Traumatic Stress Disorder after childbirth and the influence of maternity team care during labour and birth: A cohort study. Midwifery, 32(2016), 87–92. https://doi.org/10.1016/j.midw.2015.08.010

Delicate, A., & Ayers, S. (2023). The impact of birth trauma on the couple relationship and related support requirements; a framework analysis of parents’ perspectives. Midwifery, 123. https://doi.org/10.1016/j.midw.2023.103732

Delicate, A., Ayers, S., Easter, A., & McMullen, S. (2018). The impact of childbirth-related post-traumatic stress on a couple’s relationship: a systematic review and meta-synthesis. Journal of Reproductive and Infant Psychology, 36(1), 102–115. https://doi.org/10.1080/02646838.2017.1397270

Dmowska, A., Fielding-Singh, P., Halpern, J., & Prata, N. (2023). The intersection of traumatic childbirth and obstetric racism: A qualitative study. Birth, 51(1), 209–217. https://doi.org/10.1111/birt.12774

El-Salahi, S., Knowles Bevis, R., & Hogg, L. (2024). The relationship between traumatic childbirth and first-time mothers’ social identity and wellbeing: a cross-sectional observational study. BMC Pregnancy and Childbirth, 24(437), 1–11. https://doi.org/10.1186/s12884-024-06288-3

Elmir, R., Schmied, V., Wilkes, L., & Jackson, D. (2010). Women’s perceptions and experiences of a traumatic birth: A meta-ethnography. Journal of Advanced Nursing, 66(10), 2142–2153. https://doi.org/10.1111/j.1365-2648.2010.05391.x

Fenech, G., & Thomson, G. (2015). Defence against trauma: women’s use of defence mechanisms following childbirth-related trauma. Journal of Reproductive and Infant Psychology, 33(3), 268–281. https://doi.org/10.1080/02646838.2015.1030731

Garthus-Niegel, S., von Soest, T., Vollrath, M. E., & Eberhard-Gran, M. (2013). The impact of subjective birth experiences on post-traumatic stress symptoms: a longitudinal study. Archives of Women’s Mental Health, 16(1), 1–10. https://doi.org/10.1007/s00737-012-0301-3

Ghanbari-Homayi, S., Fardiazar, Z., Meedya, S., Mohammad-Alizadeh-Charandabi, S., Asghari-Jafarabadi, M., Mohammadi, E., & Mirghafourvand, M. (2019). Predictors of traumatic birth experience among a group of Iranian primipara women: A cross sectional study. BMC Pregnancy and Childbirth, 19(1), 1–9. https://doi.org/10.1186/s12884-019-2333-4

Ginter, N., Takács, L., Boon, M. J. M., Verhoeven, C. J. M., Dahlen, H. G., & Peters, L. L. (2022). The Impact of Mode of Birth on Childbirth-Related Post Traumatic Stress Symptoms beyond 6 Months Postpartum: An Integrative Review. International Journal of Environmental Research and Public Health, 19(8830), 1–26. https://doi.org/10.3390/ijerph19148830

Gottvall, K., & Waldenström, U. (2002). Does a traumatic birth experience have an impact on future reproduction? BJOG: An International Journal of Obstetrics and Gynaecology, 109(3), 254–260. https://doi.org/10.1111/j.1471-0528.2002.01200.x

Greenfield, M., Jomeen, J., & Glover, L. (2016). What is traumatic birth? A concept analysis and literature review. British Journal of Midwifery, 24(4), 254–267. https://doi.org/10.12968/bjom.2016.24.4.254

Greenfield, M., Jomeen, J., & Glover, L. (2019). “It can’t be like last time” - Choices made in early pregnancy by women who have previously experienced a traumatic birth. Frontiers in Psychology, 10(JAN), 1–13. https://doi.org/10.3389/fpsyg.2019.00056

Holopainen, A., Stramrood, C., van Pampus, M. G., Hollander, M., & Schuengel, C. (2020). Subsequent childbirth after previous traumatic birth experience: women’s choices and evaluations. British Journal of Midwifery, 28(8), 488–496. https://doi.org/10.12968/bjom.2020.28.8.488

Hüner, B., Friedl, T., Schütze, S., Polasik, A., Janni, W., & Reister, F. (2024). Post-traumatic stress syndromes following childbirth influenced by birth mode—is an emergency cesarean section worst? Archives of Gynecology and Obstetrics, 309(6), 2439–2446. https://doi.org/10.1007/s00404-023-07114-5

Ketley, R., Darwin, Z., Masterson, C., & McGowan, L. (2024). Women’s experience of post-traumatic growth following a traumatic birth: an interpretive phenomenological analysis. Journal of Reproductive and Infant Psychology, 42(1), 126–137. https://doi.org/10.1080/02646838.2022.2070608

Koster, D., Romijn, C., Sakko, E., Stam, C., Steenhuis, N., de Vries, D., van Willigen, I., & Fontein-Kuipers, Y. (2020). Traumatic childbirth experiences: practice-based implications for maternity care professionals from the woman’s perspective. Scandinavian Journal of Caring Sciences, 34(3), 792–799. https://doi.org/10.1111/scs.12786

Leinweber, J., Fontein‐Kuipers, Y., Thomson, G., Karlsdottir, S. I., Nilsson, C., Ekström‐Bergström, A., Olza, I., Hadjigeorgiou, E., & Stramrood, C. (2022). Developing a woman‐centered, inclusive definition of traumatic childbirth experiences: A discussion paper. Birth, 00, 687–696. https://doi.org/10.1111/birt.12634

Ma, D., Sun, S., Qian, J., Wang, M., Gu, H., Lou, J., & Yu, X. (2023). Predictors of pregnancy stress and psychological birth trauma in women undergoing vaginal delivery: a cross-sectional study in China. BMC Pregnancy and Childbirth, 23(596), 1–9. https://doi.org/10.1186/s12884-023-05890-1

McKelvin, G., Thomson, G., & Downe, S. (2021). The childbirth experience: A systematic review of predictors and outcomes. Women and Birth, 34(5), 407–416. https://doi.org/10.1016/j.wombi.2020.09.021

McKenzie, G. (2021). Freebirthing in the United Kingdom: The Voice Centered Relational Method and the (de)Construction of the I-Poem. International Journal of Qualitative Methods, 20, 1–13. https://doi.org/10.1177/1609406921993285

Molloy, E., Biggerstaff, D. L., & Sidebotham, P. (2021). A phenomenological exploration of parenting after birth trauma: Mothers perceptions of the first year. Women and Birth, 34(3), 278–287. https://doi.org/10.1016/j.wombi.2020.03.004

Priddis, H. S., Keedle, H., & Dahlen, H. (2018). The Perfect Storm of Trauma: The experiences of women who have experienced birth trauma and subsequently accessed residential parenting services in Australia. Women and Birth, 31(1), 17–24. https://doi.org/10.1016/j.wombi.2017.06.007

Reed, R., Sharman, R., & Inglis, C. (2017). Women’s descriptions of childbirth trauma relating to care provider actions and interactions. BMC Pregnancy and Childbirth, 17(1), 1–10. https://doi.org/10.1186/s12884-016-1197-0

Shorey, S., & Wong, P. Z. E. (2022). Traumatic Childbirth Experiences of New Parents: A Meta-Synthesis. Trauma, Violence, and Abuse, 23(3), 748–763. https://doi.org/10.1177/1524838020977161

Soet, J. E., Brack, G. A., & Dilorio, C. (2003). Prevalence and predictors of women’s experience of psychological trauma during childbirth. Birth, 30(1), 36–46. https://doi.org/10.1046/j.1523-536X.2003.00215.x

Steetskamp, J., Treiber, L., Roedel, A., Thimmel, V., Hasenburg, A., & Skala, C. (2022). Post-traumatic stress disorder following childbirth: prevalence and associated factors—a prospective cohort study. Archives of Gynecology and Obstetrics, 306(5), 1531–1537. https://doi.org/10.1007/s00404-022-06460-0

Sun, X., Fan, X., Cong, S., Wang, R., Sha, L., Xie, H., Han, J., Zhu, Z., & Zhang, A. (2023). Psychological birth trauma: A concept analysis. Frontiers in Psychology, 13(January), 1–14. https://doi.org/10.3389/fpsyg.2022.1065612

Taghizadeh, Z., Arbabi, M., Kazemnejad, A., Irajpour, A., & Lopez, V. (2015). Iranian mothers’ perceptions of the impact of the environment on psychological birth trauma: A qualitative study. International Journal of Nursing Practice, 21(S2), 58–66. https://doi.org/10.1111/ijn.12286

Taghizadeh, Z., Irajpour, A., & Arbabi, M. (2013). Mothers’ Response to Psychological Birth Trauma: A Qualitative Study. Iranian Red Crescent Medical Journal, 15(10), 1–7. https://doi.org/10.5812/ircmj.10572

Taghizadeh, Z., Irajpour, A., Nedjat, S., Arbabi, M., & Lopez, V. (2014). Iranian mothers’ perception of the psychological birth trauma: A qualitative study. Iranian Journal of Psychiatry, 9(1), 31–36.

Thomson, G., & Downe, S. (2008). Widening the trauma discourse: The link between childbirth and experiences of abuse. Journal of Psychosomatic Obstetrics and Gynecology, 29(4), 268–273. https://doi.org/10.1080/01674820802545453

Türkmen, H., YALNIZ DİLCEN, H., & Özçoban, F. A. (2021). Traumatic childbirth perception during pregnancy and the postpartum period and its postnatal mental health outcomes: a prospective longitudinal study. Journal of Reproductive and Infant Psychology, 39(4), 422–434. https://doi.org/10.1080/02646838.2020.1792429

Turkstra, E., Creedy, D. K., Fenwick, J., Buist, A., Scuffham, P. A., & Gamble, J. (2015). Health services utilization of women following a traumatic birth. Archives of Women’s Mental Health, 18(6), 829–832. https://doi.org/10.1007/s00737-014-0495-7

Tzitiridou-Chatzopoulou, M., Orovou, E., Skoura, R., Eskitzis, P., Dagla, M., Iliadou, M., Palaska, E., & Antoniou, E. (2023). Traumatic Birth Experience and Breastfeeding Ineffectiveness-a Literature Review. Materia Socio-Medica, 35(4), 325–333. https://doi.org/10.5455/msm.2023.35.325-333

Viirman, F., Hess Engström, A., Sjömark, J., Hesselman, S., Sundström Poromaa, I., Ljungman, L., Skoog Svanberg, A., & Wikman, A. (2023). Negative childbirth experience in relation to mode of birth and events during labour: A mixed methods study. European Journal of Obstetrics and Gynecology and Reproductive Biology, 282(October 2022), 146–154. https://doi.org/10.1016/j.ejogrb.2023.01.031

Watson, K., White, C., Hall, H., & Hewitt, A. (2021). Women’s experiences of birth trauma: A scoping review. Women and Birth, 34(5), 417–424. https://doi.org/10.1016/j.wombi.2020.09.016

Zhang, K., Dai, L., Wu, M., Zeng, T., Yuan, M., & Chen, Y. (2020). Women’s experience of psychological birth trauma in China: a qualitative study. BMC Pregnancy and Childbirth, 20(651), 1–8. https://doi.org/10.1186/s12884-020-03342-8
